# Identification and Characterization of Lipopolysaccharide Induced TNFα Factor from Blunt Snout Bream, *Megalobrama amblycephala*

**DOI:** 10.3390/ijms18020233

**Published:** 2017-02-15

**Authors:** Yina Lv, Xinying Xiang, Yuhong Jiang, Leilei Tang, Yi Zhou, Huan Zhong, Jun Xiao, Jinpeng Yan

**Affiliations:** 1 Department of Cell Biology, School of Life Sciences, Central South University, Changsha 410017, China; lvyina@csu.edu.cn (Y.L.); jiangyuhong@csu.edu.cn (Y.J.); tangleilei142511021@163.com (L.T.); 2 Center of Biological Experiments, School of Life Sciences, Central South University, Changsha 410017, China; bestxxy@126.com; 3 Guangxi Key Laboratory of Aquatic Genetic Breeding and Healthy Aquaculture, Guangxi Academy of Fishery Sciences, Nanning 530021, China; zhouyi1982cn@126.com (Y.Z.); zhonghuanzh@126.com (H.Z.); dreamshaw@hotmail.com (J.X.)

**Keywords:** *Megalobrama amblycephala*, lipopolysaccharide induced TNFα factor, lipopolysaccharide stimulation, innate immune

## Abstract

Lipopolysaccharide induced TNFα factor (LITAF) is an important transcription factor responsible for regulation of tumor necrosis factor α. In this study, a novel *litaf* gene (designated as *Malitaf*) was identified and characterized from blunt snout bream, *Megalobrama amblycephala.* The full-length cDNA of *Malitaf* was of 956 bp, encoding a polypeptide of 161 amino acids with high similarity to other known LITAFs. A phylogenetic tree also showed that *Malitaf* significantly clustered with those of other teleost, indicating that *Malitaf* was a new member of fish LITAF family. The putative maLITAF protein possessed a highly conserved LITAF domain with two CXXC motifs. The mRNA transcripts of *Malitaf* were detected in all examined tissues of healthy *M*. *amblycephala*, including kidney, head kidney, muscle, liver, spleen, gill, and heart, and with the highest expression in immune organs: spleen and head kidney. The expression level of *Malitaf* in spleen was rapidly up-regulated and peaked (1.29-fold, *p* < 0.05) at 2 h after lipopolysaccharide (LPS) stimulation. Followed the stimulation of *Malitaf*, *Matnfα* transcriptional level was also transiently induced to a high level (51.74-fold, *p* < 0.001) at 4 h after LPS stimulation. Taken together, we have identified a putative fish LITAF ortholog, which was a constitutive and inducible immune response gene involved in *M*. *amblycephala* innate immunity during the course of a pathogenic infection.

## 1. Introduction

Blunt snout bream (*Megalobrama amblycephala*) is one of the major economically important species in freshwater polyculture fish aquaculture in China [1]. It has been widely cultured because of its herbivorous habit, faster growth rate, and delicate flesh quality, as well as increasing demand in China during the last few decades [2,3]. In 2013, its production has reached 0.73 million tons, ranking seventh in Chinese freshwater fish production [4]. Associated with intensive farming, however, diseases caused by infectious bacteria, mainly *Aeromonas hydrophila*, frequently occur [5]. The infectious disease outbreak with quick spreading has led to serious economic losses in *M. amblycephala* culture industry. Innate immunity plays crucial roles in defense against bacterial infections in fish [6]. The innate immune response to a bacterial pathogen is characterized by the immediate release of pro-inflammatory cytokines, which act as key mediators of the immune system to eliminate the pathogen [7,8]. Finding more molecular components involved in *M. amblycephala*’s innate immunity, therefore, will facilitate our understandings in the largely unveiled complex immunity in fish.

Among the pro-inflammatory cytokines, tumor necrosis factor α (TNFα) has been confirmed to significantly trigger host immunity, increase phagocytic activity, and provoke the induction of inflammatory cytokines [9,10]. TNFα is one of the most well-known pleiotropic cytokines and is secreted by various cell types and can be regulated by different transcription factors, such as nuclear factor κB (NF-κB) [11], nuclear factor of activated T-cells (NF-AT) [12], activator protein 1 (AP-1) [13], and lipopolysaccharide induced TNFα factor (LITAF) [14]. Lipopolysaccharide induced TNFα factor (LITAF) is an important transcription factor mediating transcription of various inflammatory cytokines, especially TNFα [15]. It has been demonstrated that LITAF can directly interact with the signal transducer and activator of transcription (STAT) 6B and translocates into the nucleus where it binds to the promoter regions of TNFα and other cytokines to modulate their transcription [16]. LITAF was initially identified and characterized in the human macrophage cell line, THP-1 [17]. Since then, a large amount of LITAF homologues have been obtained in several aquatic animals including mollusk [18,19,20,21,22,23], arthropod [24,25], sea cucumber (*Apostichopus japonicus*) [26], and amphioxus (*Branchiostoma belcheri*) [27], suggesting a conserved function in innate immunity. Although the *litaf* gene has been characterized in several fish species, the knowledge of the LITAF orthologs in most teleosts is still limited [28,29,30,31].

Therefore, in the present study, we identified a novel *litaf* homolog cDNA (designated as *Malitaf*) in *M. amblycephala*, analyzed its phylogenetic relationship, and characterized its expression pattern in response to LPS stimulation. Considering that *litaf* is a vital regulator for *tnfα* expression, we therefore subsequently investigated the expression profile of *tnf*α (*Matnfα*) in *M. amblycephala*. To our knowledge, this is the first study in the LPS-induced response of *Malitaf*. The achieved results will provide a better understanding of the immune defense mechanisms and further improve the healthy management efficiency in this species.

## 2. Results

### 2.1. Isolation and Characterization of Malitaf

**Figure 1:**
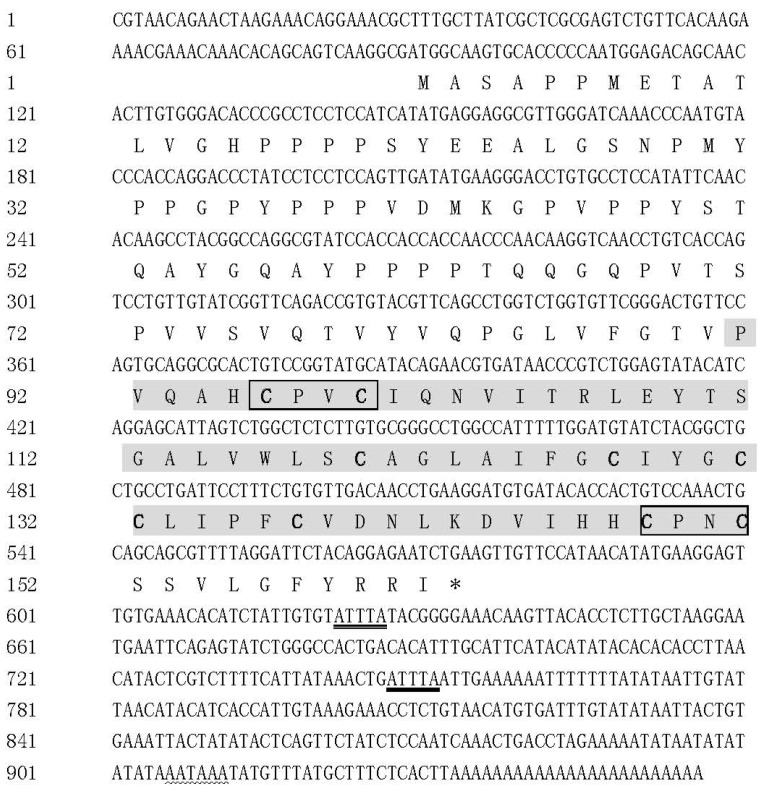
The nucleotide and deduced amino acid sequences of *Malitaf*. The predicted lipopolysaccharide induced TNFα factor (LITAF) domain is shaded. The two CXXC motifs are in shaded boxes, and the cysteine residues are indicated in shaded bold face. The ATTTA and AATAAA are double and wavy underlined, respectively. “*” shows stop codon.

The complete *Malitaf* cDNA sequence was 956 bp, which was composed of an 88-bp length 5′-untranslated region (5′-UTR), a 486-bp open reading frame encoding a protein comprising 161 amino acids, and a 358-bp 3′-UTR followed by a poly (A) tail (Figure 1). One putative polyadenylation signal (AATAAA) was recognized at the nucleotide position 906, which was 21 nucleotides upstream of the poly (A) tail. Furthermore, there were two cytokine RNA instability motifs (ATTTA) at the 3′-UTR of *Malitaf*, which were also presented in the *litaf* gene of *Paralichthys olivaceus* [28]. This cDNA sequence has been deposited in the GenBank database under accession number KX421367.

**Figure 2:**
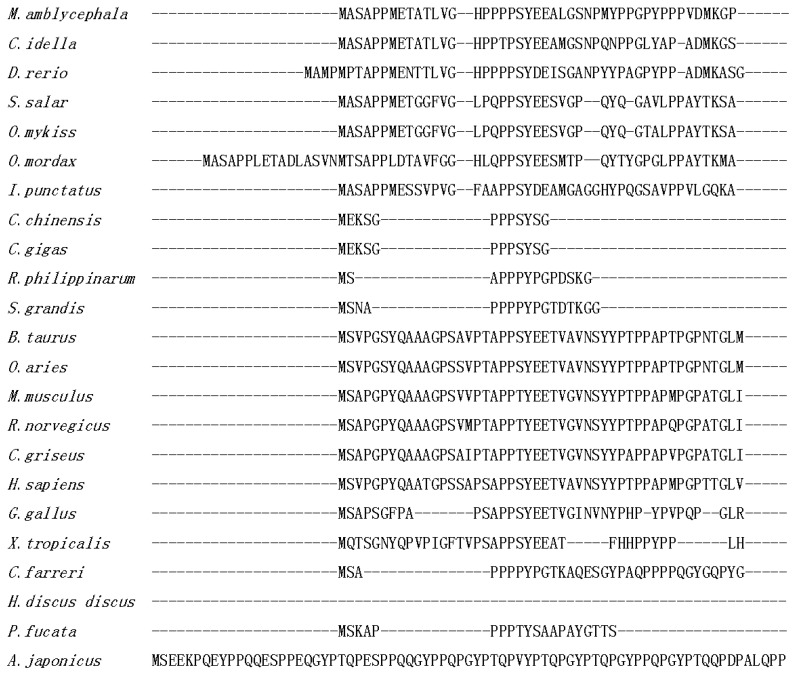

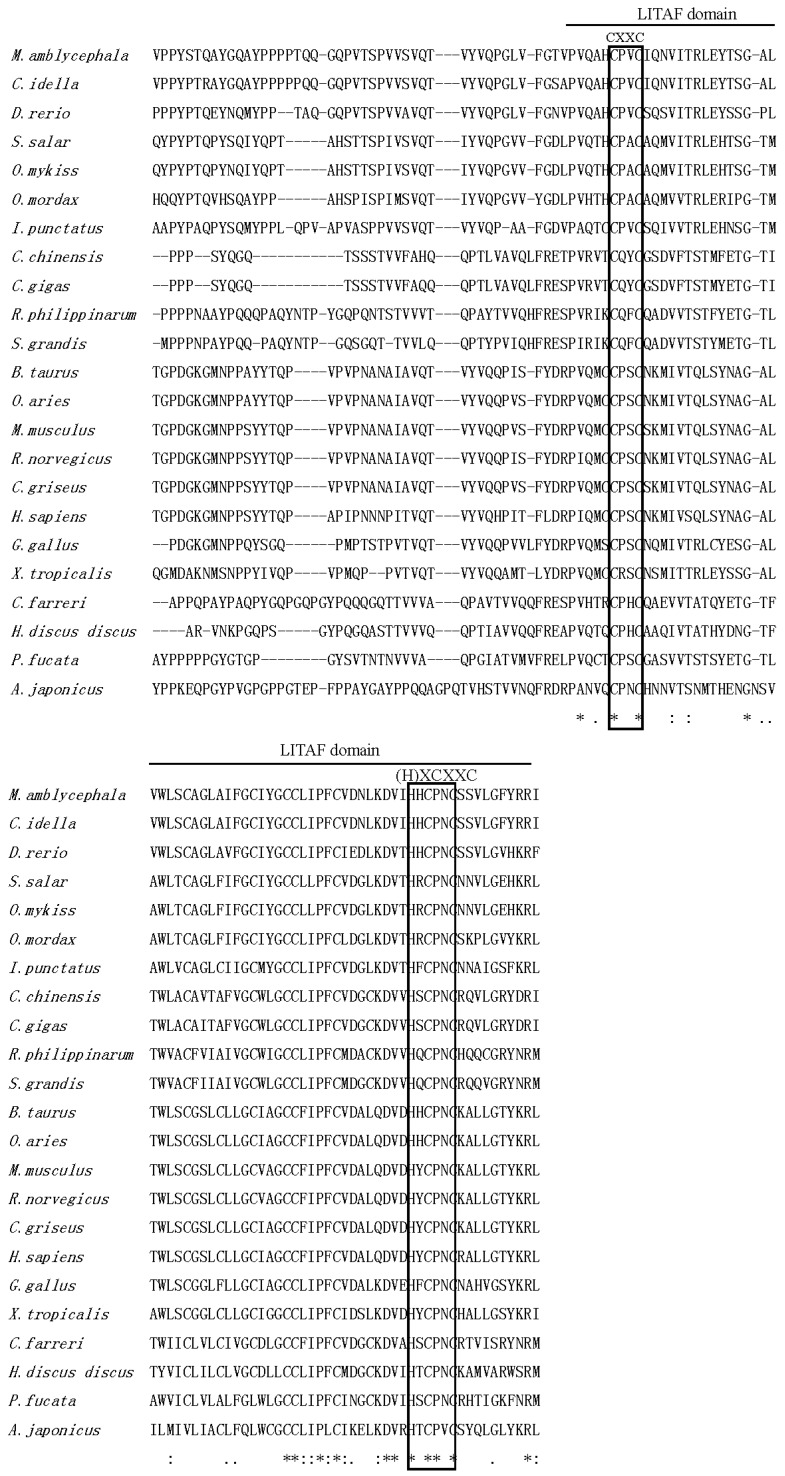
Multiple sequence alignment of LITAFs. The identical, highly conserved, and less conserved amino acid residues are indicated by “*”, “:”, and “.”, respectively. The gaps in the alignment are indicated by “-”. The LITAF domain was labeled above the sequences, and two motifs were indicated with rectangles.

The deduced protein from *Malitaf* gene (MaLITAF) possessed with an estimated molecular mass of 17.2 kDa and an isoelectric point of 6.00. The protein analysis by Basic Local Alignment Search Tool (BLAST) showed that MaLITAF protein shared the highest identity (91.3%) with that of grass carp (*Ctenopharyngodon idellus*). Multiple alignment of amino acid sequences of LITAFs from different species revealed that the C-terminal region of the MaLITAF showed much higher homology than that of other regions (Figure 2). Furthermore, similar to other LITAF homologues, the maLITAF protein possessed the typical LITAF domain (91–160 aa) that contained an N-terminal CXXC “knuckle” followed by a long hydrophobic region, and a C-terminal (H)XCXXC knuckle (Figure 2). The CXXC and HXCXXC motifs are highly conserved among invertebrates, mammals, and fish.

**Figure 3:**
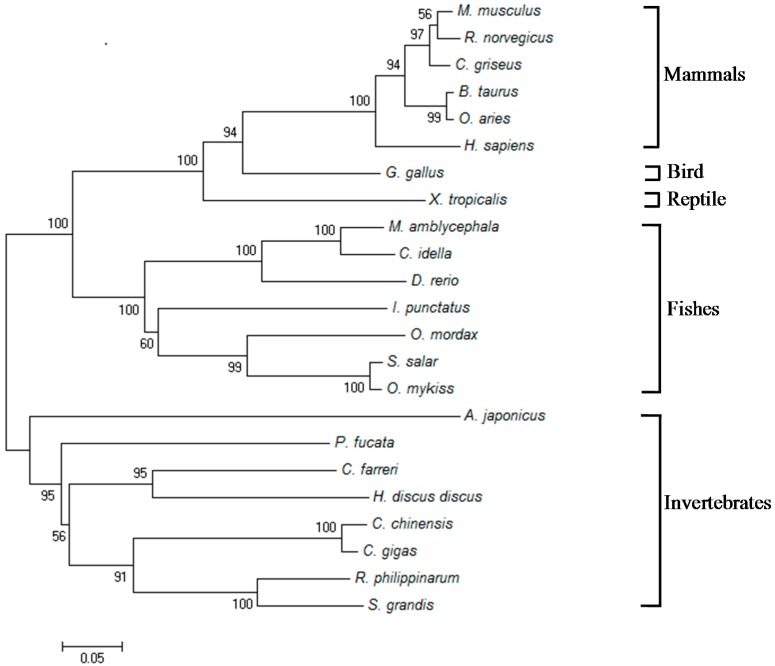
The neighbor-joining phylogenetic tree of MaLITAF protein and other known homologues from GenBank registered. The numbers at the nodes represent bootstrap values for 1000 replications and the bar (0.1) indicates genetic distance. The sequences of LITAFs from other species were downloaded from GenBank: *C*. *idella* (ACT68335), *Danio rerio* (NP_001002184), *Ictalurus punctatus* (NP_001187935), *Oncorhynchus mykiss* (NP_001158593), *Osmerus mordax* (ACO09164), *Salmo salar* (ACI67257), *Gallus gallus* (NP_989598), *Xenopus tropicalis* (NP_988970), *Mus musculus* (NP_064364), *Homo sapiens* (NP_004853), *Bos taurus* (NP_001039717), *Cricetulus griseus* (XP_003496739), *Ovis aries* (XP_004020821), *Rattus norvegicus* (NP_001099205), *Solen grandis* (AEW43450), *Ruditapes philippinarum* (ADX31291), *Crassostrea gigas* (ABO70331), *Cipangopaludina chinensis* (AEX08893), *Haliotis discus discus* (ADI72430), *Chlamys farreri* (ABI79459), *Pinctada fucata* (ACN70008), and *A. japonicus* (AIB51692).

A phylogenetic tree was constructed by the neighbor-joining method based on entire amino acid sequences of LITAFs from *M. amblycephala* and other species. As shown in Figure 3, all LITAFs were split into five categories, including mammalian, avian, amphibian, teleost, and invertebrate LITAFs. Obviously, *Malitaf* was located into the fish LITAF group which was distinct from the mammalian cluster. As expected, *Malitaf* showed the closest relationship to *C. idellus*. The results indicated that *Malitaf* represents a new member of fish LITAF family.

### 2.2. Tissues Distribution of Malitaf

**Figure 4:**
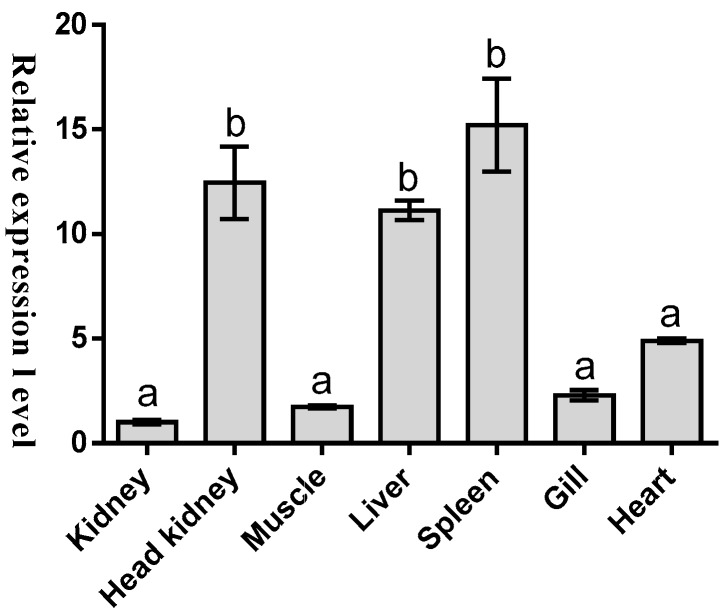
The expression of *Malitaf* mRNA in different tissues from healthy *M. amblycephala*. Significant pairwise expression-level differences between tissues are indicated by different letters above the bars.

To further understand the potential function of this new gene, the presence of *Malitaf* mRNA transcript in different tissues from healthy *M. amblycephala* was examined by quantitative real-time PCR (qRT-PCR) analysis, which can accurately quantify transcripts at a low copy number [32]. Expression was normalized to the tissue with the lowest observed mRNA level-kidney (set as 1). As shown in Figure 4, the mRNA transcript of *Malitaf* was ubiquitously detected in a wide range of tissues examined from healthy fish. However, the relative gene expression of *Malitaf* to kidney was the highest in spleen (15.21-fold, *p* < 0.001), which was also observed in spleen of rock bream (*Oplegnathus fasciatus*) [30], followed by head kidney (12.45-fold). The transcriptional level of *Malitaf* decreased gradually in liver (11.12-fold), heart (4.9-fold), gill (2.29-fold), and muscle (1.75-fold).

### 2.3. Temporal Expression of Malitaf and Matnfα after LPS Stimulation

**Figure 5:**
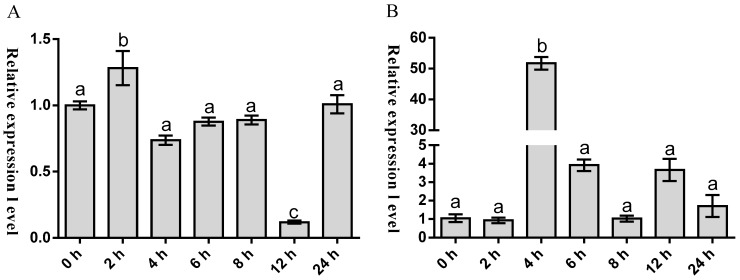
Temporal expression analysis of *Malitaf* (**A**); and *Matnfα* (**B**) mRNA in spleen from *M. amblycephala* after LPS stimulation. Significant pairwise expression-level differences among different time-points are indicated by different letters above the bars.

To preliminarily unravel the potential role of *Malitaf* in innate immunity, we characterized the temporal expression pattern of *Malitaf* in spleen in response to stimulation with LPS. As shown in Figure 5A, the mRNA expression of *Malitaf* was rapidly up-regulated after LPS stimulation. The expression reached a peak level at 2 h post-stimulation, which was about 1.29-fold higher than control group (*p* < 0.05). Subsequently, it was found that its expression decreased gradually to the control level as time elapsed. However, a significant decrease of *Malitaf* expression was observed at 12 h after LPS stimulation. Considering that *litaf* is an important regulator for *tnfα* expression, we therefore tested the expression profile of *Matnfα* following the elevated expression of *Malitaf* under LPS stimulations at different time points. As shown in Figure 5B, the transcription of *Matnf* maintained the control level at 2 h post LPS stimulation. However, a sharp increase of *Matnf*α mRNA to peak level was detected at 4 h post LPS stimulation (51.74-fold, *p* < 0.001), and then dropped to the original level at 8 h post LPS stimulation. Interestingly, the transcription of *Matnf*α surged again to a higher level at 12 and 24 h post LPS stimulation (3.66- and 1.71-fold, respectively) compared with that of the control group.

## 3. Discussion

Cytokines are the key regulators of innate immunity against pathogens [33]. Until now, *litaf* gene had been cloned and characterized from many organisms including both vertebrate and invertebrate animals, which suggested a conserved function in innate immunity. In mammals, the *litaf* has been reported as an important transcript factor for the regulation of TNFα and transcription of various inflammatory cytokines in mammals [34]. However, the information on the systemic reaction of fish *litaf*s during bacterial infection was rather rare. Therefore, we cloned the novel *litaf* gene from blunt snout bream and examined their expression profiles to understand its potential role in innate immunity.

The LITAF domain, which is a key feature for the LITAF family, exists in viruses, fungi, plants, and Metazoa, [35]. In the present study, the amino acid alignment indicated a highly conserved LITAF domain with N-terminal CXXC and C-terminal HXCXXC knuckles that formed a compact Zn^2+^-binding structure. These characteristics are a key feature of intracellular Zn^2+^-binding domains that the N-terminal region binds to the intracellular molecule and the hydrophobic region does not span the membrane [36]. These observations indicated that the LITAF domain of *Malitaf* appears to be capable of responding similarly to the mammalian LITAF. In a phylogenetic tree of selected vertebrate and invertebrate LITAF amino acid sequences, the MaLITAF was obviously separated from the mammalian cluster and formed one distinct cluster with other teleost LITAFs, suggesting that it is a fish-specific LITAF.

We performed qRT-PCR to monitor the transcriptional level of *Malitaf* in various tissues from the healthy fish. The results showed the constitutive distribution of *Malitaf*, indicating that its important role in immune defense against invaders and its immune responses could be occurring in the whole fish body. In teleost fish, spleen and head kidney could be considered as the important central immune organs synthesizing proteins involved in fish immune defense [37]. Therefore, the higher expression level of *Malitaf* in spleen and head kidney also particularly suggested its crucial role in immune defense against potential pathogens. In other teleost fish, the constitutive expression of *litaf* mRNA has been reported in a wide range of tissues, but with varying expression levels. For example, the relative gene expression of *litaf1* of rock bream was the highest in spleen. In Japanese flounder, the *litaf* mRNA was detected in all examined tissues with the greatest amount in gill, followed by blood, skin, gonad, hepatopancreas, head kidney, heart, brain, trunk kidney, and spleen in a descending order [28]. The grouper *litaf* gene was also widely expressed in different tissues analyzed, including liver, spleen, kidney, head kidney, intestine, skin, gill, brain, muscle, heart, and stomach [29]. However, the relatively low expression levels were detected in muscle and liver. In grass carp, the *litaf* gene was found to express in various tissues but with a high expression level in gill [31]. The inconsistent constitutive expression of *litaf* gene among different fish species may be a reflection of physiological differences among these species, or even environmental influences. Furthermore, the differences in *litaf* gene expression are thought to be possibly due to different expressing cells and functions of *litaf* gene. Certainly, further studies on functional differentiation of *litaf* gene between fish and other species may yield more novel information on the immune regulatory response of fish.

The *Malitaf* expression was significantly induced in spleen by LPS, a compound that mimics a Gram-negative bacteria infection, which was similar to the previous reports on human THP-1 cells [17], mouse [38], chicken macrophages [39], Pacific oyster [22], and scallop hemocytes [23]. The results strongly suggested its significantly responsive to LPS and involvement in innate immune response against Gram-negative bacteria. After 12 h of LPS stimulation, the *Malitaf* mRNA has significantly decreased, which might possibly prevent the excess production of cytokines, and then the innate immune response to pathogens could be efficiently controlled [33]. The expression pattern of *Malitaf* appeared to be similar to Japanese flounder *litaf* with respect to the expression profile in response to LPS stimulation [28]. TNFα effects can be both beneficial and detrimental to the host [40]. TNFα must be tightly regulated because over-production can be lethal to the host as in septic shock syndrome. The mouse *litaf* gene induces activation of *tnfα* gene expression by itself [38]. Inhibiting human *litaf* expression in a human monocytic cell line leads to a reduction in the *tnfα* transcript [15]. Therefore, in the present study, we tested the expression profile of *Matnfα* following the elevated expression of *Malitaf* under LPS stimulation. The *Matnfα* was also induced and rapidly peaked at 4 h, not at 2 h, post LPS administration indicating that *Malitaf* may significantly contribute to the up-regulated *Matnfα* expression in the spleen. In addition, the associated up-regulation of *Malitaf* and *Matnfα* in spleen during the early phase of LPS stimulation process indicated that *Malitaf* may be an essential regulator involved in *M. amblycephala* innate immune response, probably through regulation of *Matnfα* expression.

## 4. Materials and Methods

### 4.1. Fish, LPS Stimulation, and RNA Isolation

Adult *M. amblycephala* (weight: 405 ± 12.4 g) were obtained from a fish farm (Changsha, China). Fish were maintained with a flow-through water supply at room temperature. After acclimating for one week, the normal fish were used for the stimulation experiments. LPS isolated from *Escherichia coli* (L2880, Sigma, St. Louis, MO, USA) was suspended into sterilized phosphate buffered saline (PBS), and then was intraperitoneally injected into fish at a dose of 0.1 mg/100 g fish. Afterwards, fish were anesthetized with MS-222 (3-aminobenzoic acid ethyl ester; Sigma). Various tissues from three healthy individuals, including kidney, head kidney, muscle, liver, spleen, gill, and heart, were collected. Similarly, the spleen from three individuals was collected at different time points (0, 2, 4, 6, 8, 12, and 24 h) after stimulation. Total RNA from above tissues was extracted using TRIzol reagent (Invitrogen, Carlsbad, CA, USA), and quantified based on the absorbance at 260 nm. The integrity of RNA was checked by agarose gel electrophoresis. The animal experiments were approved by the Ethics Committee of School of Life Sciences of Central South University with the following reference number (SLSEC0028) in 10 March 2015. The tissues collected at 0 h were from fish injected with the same volume of PBS.

### 4.2. Cloning and Characterization of Malitaf

To obtain the partial cDNA of *Malitaf*, the degenerate primers were designed based on an alignment of its counterparts from other teleost fish (Table 1). Gene-specific primers were used to amplify each end according to the manufacturer’s instructions. All resulting PCR products were purified and then cloned into the pMD18-T vector (TaKaRa, Dalian, China) and sequenced bi-directionally at Sangon (Shanghai, China). The open reading frame (ORF) of *Malitaf* cDNA was detected using the ORF finder (available on: http://www.ncbi.nlm.nih.gov/project/gorf). Protein domains were predicted by the Simple Modular Architecture Research Tool (SMART) [41]. Multiple sequence alignments were created using the Clustal W program [42]. Phylogenetic and molecular evolutionary analyses were constructed by the Neighbor-Joining method in Molecular Evolutionary Genetics Analysis (MEGA) software (version 7.01, Tokyo Metropolitan University, Tokyo, Japan), and support for each node was bootstrapped with 1000 replicates [43].

### 4.3. Spatial and Temporal Expression Analysis of Malitaf and Matnfα

The quantitative real-time PCR (qRT-PCR) was performed to investigate *Malitaf* mRNA expression levels in different tissues of healthy *M. amblycephala*. In addition, the mRNA expression pattern of *Malitaf* and *Matnfα* was determined in spleen after LPS stimulation by qRT-PCR. The β-actin and 18S rRNA was selected as an internal control to verify the successful reverse transcription and to calibrate the cDNA template in spatial and temporal expression analysis, respectively. The qRT-PCR was implemented using an ABI 7500 Real-time PCR system (Applied Biosystems, Foster, CA, USA) in a total volume of 20 µL, including 10 µL SYBR^®^ Premix Ex Taq™ II (2×) (TaKaRa, Dalian, China), 0.4 µL ROX Reference Dye II (50×), 0.4 µL of each primer (10 µmol∙L^−1^), 2 µL 1:5 diluted cDNA, and 6.8 µL of PCR-grade water. The thermal profile was 95 °C for 30 s followed by 40 cycles of 95 °C for 5 s, 60 °C for 34 s, and 72 °C for 30 s. Melting curve analysis of the amplified products was performed at the end of each PCR to confirm that a single PCR product was generated. The 2^−∆∆*C*t^ method was used to analyze the expression levels of *Malitaf* and *Matnfα* genes [32]. The data obtained from three independent biological replicates were subjected to statistical analysis and the values represented the *n*-fold difference relative to the references (kidney and 0 h).

### 4.4. Statistical Analysis

All data of qRT-PCR were presented as means ± SD and checked for homogeneity of variances and normality. Statistical analysis was performed using GraphPad Prism 5.0 (GraphPad Software Inc., San Diego, CA, USA). Significant differences among samples were determined by one-way analysis of variance (one-way ANOVA) followed by a Tukey’s multiple comparison test. Differences were considered significant at *p* < 0.05 and extremely significant at *p* < 0.01.

## 5. Conclusions

We identified and characterized a new member of LITAF family, *Malitaf*, in *M. amblycephala*. We confirmed that *Malitaf* mRNA constitutively expresses in all examined tissues and displays a higher expression level in spleen and head kidney than in other tissues. We also demonstrated that *Malitaf* expression could be induced by bacterial endotoxin LPS stimulation. Furthermore, we showed that the expression of *Matnfα*, a pleiotropic cytokine regulated by *Malitaf*, was up-regulated associated with the enhanced expression of *Malitaf* in vivo. Our results significantly suggested that *Malitaf* may play a key role in blunt snout bream innate immunity. Further studies on the functions of *Malitaf* will contribute to a better understanding of the fish immune system and may help elucidate fish immunoregulatory pathways.

## Figures and Tables

**Table 1:** Sequences of primers used in this study.

Primer	Sequence(5′–3′)	Comment
LITAF-F	AARCGCTTTGYTTRTCGCTC	Gene cloning
LITAF-R	AGGTGYAAMTTGTTTCCCCG	
3′-Adaptor primer	GCTGTCAACGATACGCTACGTAACGGCATGACAGTG(T)_18_	3′RACE
3′-Primer	GCTGTCAACGATACGCTACGTAACG	
3′-Nested primer	CGCTACGTAACGGCATGACAGTG	
LITAF-3′-GSP	ACAGCAACACTTGTGGGACACCCGCCT	
LITAF-3′-NGSP	AGCCTGGTCTGGTGTTCGGGACTGTT	
AAP	GGCCACGCGTCGACTAGTACGGGIIGGGIIGGGIIG	5′RACE
AUAP	GGCCACGCGTCGACTAGTAC	
LITAF-5′-GSP	CTGTGCCTCCATATTCAACACAAG	
LITAF-5′-NGSP	GTTGGGATCAAACCCAATGTACC	
LITAF-qF	CACCAGTCCTGTTGTATCGG	Real-time PCR
LITAF-qR	CGCACAAGAGAGCCAGACTA	
TNFα-qF	CTGCTGTCTGCTTCACGCTC	
TNFα-qR	TAAATGGATGGCTGCCTTGG	
β-actin-qF	CGGACAGGTCATCACCATTG	
β-actin-qR	CGCAAGACTCCATACCCAAGA	
18S rRNA-qF	CAAGACGGACGAGAGCGAAA	
18S rRNA-qR	GCGGGTTGGCATAGTTTACG	
